# Mixed methods inquiry into traditional healers’ treatment of mental, neurological and substance abuse disorders in rural South Africa

**DOI:** 10.1371/journal.pone.0188433

**Published:** 2017-12-19

**Authors:** Carolyn M. Audet, Sizzy Ngobeni, Erin Graves, Ryan G. Wagner

**Affiliations:** 1 Vanderbilt Institute for Global Health, Vanderbilt University, Nashville, United States of America; 2 MRC/Wits Agincourt Research Unit, School of Public Health, Faculty of Health Sciences, University of Witwatersrand, Johannesburg, South Africa; 3 Umeå Centre for Global Health Research, Umeå University, Umeå, Sweden; Yale University Yale School of Public Health, UNITED STATES

## Abstract

**Background:**

Traditional healers are acceptable and highly accessible health practitioners throughout sub-Saharan Africa. Patients in South Africa often seek concurrent traditional and allopathic treatment leading to medical pluralism.

**Methods & findings:**

We studied the cause of five traditional illnesses known locally as “*Mavabyi ya nhloko*” (sickness of the head), by conducting 27 in-depth interviews and 133 surveys with a randomly selected sample of traditional healers living and working in rural, northeastern South Africa. These interviews were carried out to identify treatment practices of mental, neurological, and substance abuse (MNS) disorders. Participating healers were primarily female (77%), older in age (median: 58.0 years; interquartile range [IQR]: 50–67), had very little formal education (median: 3.7 years; IQR: 3.2–4.2), and had practiced traditional medicine for many years (median: 17 years; IQR: 9.5–30). Healers reported having the ability to successfully treat: seizure disorders (47%), patients who have lost touch with reality (47%), paralysis on one side of the body (59%), and substance abuse (21%). Female healers reported a lower odds of treating seizure disorders (Odds Ratio (OR):0.47), patients who had lost touch with reality (OR:0.26; p-value<0.05), paralysis of one side of the body (OR:0.36), and substance abuse (OR:0.36) versus males. Each additional year of education received was found to be associated with lower odds, ranging from 0.13–0.27, of treating these symptoms. Each additional patient seen by healers in the past week was associated with roughly 1.10 higher odds of treating seizure disorders, patients who have lost touch with reality, paralysis of one side of the body, and substance abuse. Healers charged a median of 500 South African Rand (~US$35) to treat substance abuse, 1000 Rand (~US$70) for seizure disorders or paralysis of one side of the body, and 1500 Rand (~US$105) for patients who have lost touch with reality.

**Conclusions:**

While not all healers elect to treat MNS disorders, many continue to do so, delaying allopathic health services to acutely ill patients.

## Introduction

Mental health, neurological, and substance abuse disorders (MNS) are the leading cause of disability worldwide, accounting for 28.5% of all years lived with disability[1] Despite this global burden, fewer than 25% of affected individuals ever access mental health or neurological treatment [2]; in low and middle income countries (LMICs), utilization is much lower [3]. In sub-Saharan Africa (SSA), MNS are largely neglected, misdiagnosed, or mistreated [4]. The process of integrating mental health services into primary health care has been delayed in part by constraints in health infrastructure in rural areas, and service availability is usually limited to tertiary psychiatric facilities [5,6]. In South Africa, 16.5% of adults suffered from a common mental disorder (anxiety, depression, substance abuse) in the past year; only 5.6% of those with a disorder received allopathic health treatment [7]. MNS services are now being introduced in primary care facilities in an effort to engage patients with lower incomes and those living in rural areas, yet integration is slow [4]. The recognition of the paucity of allopathic mental health care providers in South Africa’s public sector—there are only 0.4 psychiatrists and 0.3 psychologists per 100,000 people [8]–has led to innovative solutions, including task sharing models being proposed and recently adopted in the government’s Mental Health Policy Framework and Action Plan (2013–2020)[9]. However, even with these innovative policies, challenges in implementing the integration remain [10].

It is widely believed in SSA that traditional healers have the unique ability to diagnose and treat physical and emotional conditions brought about by such causes as social misconduct, spirits, spells, and sorcery, which allopathic clinicians lack the ability to treat [11–14]. Additionally, healers typically speak patients’ preferred local languages, are often living closer to and more readily available to attend to patients, and allocate more time to explain diagnoses, causes of illnesses, and treatments with patients [15]. Traditional healers may be able to provide therapy and services that allopathic health systems are not able to provide. Motivated by these factors, as well as limitations amidst the allopathic workforce and infrastructure, patients experiencing a variety of acute and chronic mental and physical health concerns often frequent a traditional healer instead of, or in conjunction with, seeking allopathic services [15–19]. This type of medical pluralism, combined with the present divide between the traditional and allopathic systems, results in patients “ping-ponging” between the two systems, often receiving insufficient care to sustainably improve their health and well-being [7,17]. Among patients who do receive mental health services, few receive counseling or support for long-term treatment adherence. Widespread social stigma surrounding mental health illness and lack of perceived appropriate biomedical care results in avoidance of health-seeking behaviors for suspected MNS disorders [20,21]. Little community outreach is done to educate affected families. These barriers often lead to delayed, intermittent or abandoned biomedical care resulting in increased morbidity and mortality [1,14,22–29]. Traditional healers, given their respected position within the community, physical proximity to patients and cultural understanding may be able to overcome a number of these traditional barriers to support the provision of integrated mental health care.

Among South Africans with a chronic DSM-IV diagnosis in 2009, 29% reported seeking allopathic care, 9% sought traditional care, and 11% saw a spiritual or religious advisor. Predictors for seeking traditional care were older age, black race, lower education level, unemployment, and experiencing anxiety or substance abuse issues [30]. Patients continue to seek traditional care for MNS ailments, and healers continue to treat patients for these disorders [30,31]; however, there is a paucity of information available regarding the frequency and types of treatments for MNS illnesses offered by traditional healers working in South Africa. More information is needed concerning which MNS conditions healers elect to treat, beliefs about disease etiology, treatment methods undertaken, and the cost for treatment. We conducted this mixed-methods study with a sample of local traditional healers to investigate which MNS conditions traditional healers treat and the cost of treatment as well as the traditional healers’ willingness to work with the allopathic health care system.

## Methods

### Study location

We conducted this study in the sub-district of Agincourt located in the Bushbuckridge area of Mpumalanga province in South Africa, roughly 500 km northeast of Johannesburg. The MRC/Wits Agincourt Research Unit (www.agincourt.co.za) manages the Agincourt Health and Socio-Demographic Surveillance Site (HDSS), which since 1992 has continuously undertaken population-based health and demographic research [32–34]. The Agincourt HDSS population is made up of approximately 115,000 residents living across roughly 21,000 households within 31 research villages. About one-third of the HDSS population, which is mainly xi-Tsonga-speaking, is made up of Mozambican refugees (or descendants of) who had immigrated to South Africa in the 1980s. The research site includes seven government-run health clinics (providing free, basic outpatient care, including immunization, family planning, testing and treatment for sexually transmitted infections, minor trauma and chronic disease care), one public-private community health center (focusing on HIV and tuberculosis treatment as well as chronic non-communicable disease treatment), and one sizeable public health center, which provides 24-hour maternity care as well as acute care and 48-hour patient observation. Patients seen at the health centers are referred, when needed, to three district hospitals, each located roughly 25–55 km from the site, generally requiring public transport for travel.

### Study population

We used this registration list of the Kukula Traditional healer organization to randomly sample participants through the random selection function in Stata® (StataCorp, LP, College Station, Texas, USA).[35] The Kukula Traditional Healer organization is an organization of roughly 300 registered traditional healers who work within the Bushbuckridge area and is the only organized group of healers living in the region. Out of a total of 280 registered healers, 169 were randomly selected to participate (using Stata® 13); of these four no longer lived in the research area, and five had since died (95% response rate). Each of the remaining 160 healers who were approached accepted to participate in the study (i.e., 100% acceptance rate). We conducted qualitative interviews until data saturation around attitudes towards mental health treatment practices was reached. We enrolled 133 participants in the quantitative portion of our study to ensure we could estimate (with 95% confidence) the proportion of healers who treated HIV (the focus of a related study with the same population of healers) with a precision of 6%.

### Data collection

With the random sample of traditional healers from the Bushbuckridge area we conducted 27 qualitative interviews and 133 surveys between July 2014 and August 2015. In-depth interviews (IDIs) were completed first in order to gather data regarding the illnesses healers treated, which they felt they had treated most effectively, causes of those illnesses, their preferred treatment methods, service fees charged to patients, and their thoughts about the ability of allopathic medicine to treat these illnesses. Specifically, healers described the symptoms they could effectively treat or cure, and were asked the xi-Tsonga and English word for that illness (if known). All interviews and surveys were conducted using the xi-Tsonga disease name identified by the healers during qualitative interviews.

The IDIs were conducted in the language the traditional healer was most conversant in (mainly xi-Tsonga) in the location of the traditional healer’s choosing (generally, in the traditional healer’s home or place of work) by a trained qualitative study team member (SN). Interviews lasted an average of 61 minutes during which we asked healers, what types of traditional mental illnesses they treated, the symptoms associated with each illness, what treatments they could provide, patient prognosis, and what, if any, allopathic diagnosis they associated with the traditional diagnosis.

Based on findings from the IDIs, we developed quantitative surveys to determine which illness(es) healers treated, their thoughts on the effectiveness of treatments they provide, and fees charged. MNS-specific illnesses focused on included: (1) seizure disorders; (2) patients who have lost touch with reality; (3) paralysis on one side of the body, and (4) substance abuse (drug and alcohol abuse). Data was also gathered regarding the number and type of patient diagnoses within the previous seven and 30-day time periods.

### Data availability

Data are available on the Harvard dataverse at http://dx.doi.org/10.7910/DVN/TAJKBK.[36]

### Data analysis

Participant characteristics, service provision, and cost per service were presented as frequencies with percentages, means, and medians with interquartile ranges (IQR). Multivariable logistic regression was used to model the odds of treating select MNS disorders, including seizure disorders, patients who have lost touch with reality, substance abuse, and paralysis on one side of the body, which is often associated with stroke. All regressions reported in this study were adjusted for age, level of education, immigrant status, number of patients per month, and type of healer.

Within two weeks of conducting the interview the qualitative data were transcribed into the local language and were then translated into English (as needed). Two research team members with considerable experience conducting qualitative research completed the thematic analysis using MAXQDA® (VERBI GmbH, Berlin, Germany) software. Coding was carefully reviewed for agreement; comparison of coding for the first five interviews found 90% agreement using Cohen’s Kappa in MAXQDA®. Any incongruities in coding were resolved through consensus.

### Ethical approvals

Approval was received for this study from the Vanderbilt Institutional Review Board (IRB # 140646) and the University of Witwatersrand Institutional Review Board (IRB #140547). All study participants provided written informed consent.

## Results

### Traditional healer demographics

Healers sampled from the rural Bushbuckridge area were predominantly female (77%), older (58 years; interquartile range [IQR]: 50–67), had long-term experience practicing traditional medicine (17 years; IQR: 9.5–30), and had low formal education levels (3.7 years; IQR: 3.2–4.2). Ninety-two healers (69%) self-identified primarily as an Inyanga (herbalist) healer, while 34 (26%) self-identified as a Sangoma (divine treatment) healer, and 5% identified as “other”. Participating healers who were actively treating patients at the time of the study reported seeing a median of three patients in the previous week. Slightly more than half the responding healers reported no association with any particular religion (n = 68, 52%), while the most common self-identified affiliations were Zion (n = 19, 15%), Apostolic (n = 11, 9%), and Bandla Lama Nazaretha (n = 10, 8%). Healers were asked if they, their parents and/or grandparents had emigrated from Mozambique, to which 94 (71%) responded positively; however, only 4 (3%) reported fluency in Portuguese. Healers reported their most commonly spoken languages as: xi-Tsonga (n = 130, 98%), isiZulu (n = 40, 30%), English (n = 12, 9%), and Xhosa (n = 8, 6%).

### Diagnosis and treatment

#### Perceived ability to diagnosis and treat

Healers claimed the ability to successfully treat the following MNS illnesses: seizure disorders (47%), patients who had lost touch with reality (47%), patients with depressive symptoms (22%), patients with paralysis on one side of the body (59%), and substance abuse (21%) (Table 1). Collectively, healers reported that in the month prior to their survey interview, they treated two adults and three children with seizure disorders, five adults and one child who had lost touch with reality, two adults who had experienced paralysis on one side of the body, and six adults who were ‘possessed by an evil spirit’. In addition, healers provided medications to seven infants to prevent the development of seizure disorders.

**Table 1:** Percentages of healers treating MNS disorders and associated costs.

	% Treat	Cost (median, IQR)*	Cost (mean, SD)*
Seizure Disorder (children)	63 (47.4%)	1000 (500, 1500)	1212 (963)
Seizure Disorder (adults)	62 (46.6%)	1000 (500, 1500)	1212 (963)
Lost touch with reality (children)	58 (43.6%)	1500 (1000, 2500)	1915 (1336)
Lost touch with reality (adults)	63 (47.4%)	1500 (1000, 2500)	1915 (1336)
Paralysis on one side of the body (adults)	78 (58.6%)	1000 (500, 1500)	1247 (1088)
Substance abuse (children)	17 (12.8%)	500 (250, 1500)	1326 (2808)
Substance abuse (adults)	28 (21.1%)	500 (250, 1500)	1326 (2808)

*All costs are reported in South African Rand (US$1 = ~14 Rand).

Many interviewees alluded to a long-standing history of healers treating mental illness and related disorders; as one healer explained, “*It is a natural illness and even in the past the traditional healers were treating patients with mental illness and even now they are still treating them and they get cured*” (male, 59 years). Some healers acknowledge that they are able to diagnose and know the cause of their patients’ illnesses without testing: “*we have been given that gift as seer*” (female, 54 years). For others, the possibility of curing a mental illness depends on how long the patient has had symptoms, for example, “*if … it has started last month or two months back*, *I will cure that person but if it’s after some years*, *no I will not able to cure that person because the illness is very strong in his body*” (male, 31 years). Further still, not all healers are confident in their ability to treat, or may lack any desire to attempt to treat mental illness or MNS disorders; as one healer revealed, “*I am scared of people with mental illness … my father was treating people with mental illness and when he try to teach me I said no*, *I don’t want to do this*” (female, 74 years). Some healers intimated that certain mental illnesses cannot be cured: “*What I can tell you is that mental illness is incurable; there are times when it will come again*” (female, 43 years), but may be controlled with recurrent traditional treatments: “*When you have mental illness you have to go to the healer who treated you after a year so that he can give you the treatment again*” (male, 67 years).

#### Seizure disorders (xi-Tsonga name: *Mavabyi ya ku wa*)

Forty-seven percent of healers believed they could successfully treat adults experiencing Mavabyi Ya Ku Wa. There was strong agreement about the symptoms and cause of seizure disorders. On healer explained “It’s a snake that causes it, you find that the snake that is inside turn and it cause him to have seizure. People live with many organs in their body, there is a snake that allows food to enter and there is a snake that gives life; the one that allow food is working together with the one in the brain. If the snake can turn and look down and then it will cause seizure to a person… When a person has the illness he will start by looking like he is lonely, after that he will have seizure.” (male, 67 years) The snake is also thought to cause seizures in children, although the symptoms were described slightly differently. “When the child is born you find that she has hiccups and most of the time and it is a snake that is inside her that makes the hiccups, when the snake starts you will hear the sound inside and the child will vomit in most of the time. Then you will take the child to the hospital to consult, and by the time you are doing that the snake is growing inside the child. And when she grow up and stop breastfeeding you find that she starts to have seizure and collapse. It caused by a snake” (female, unknown age). Many healers conveyed confidence in treating seizure disorders, particularly in children. Treatment options included a mixture of trees given in a tea, the mixture of herbs mixed with porridge, and mixing a patient’s blood with that of a pig. Still, some healers express hesitation to treat seizure disorders, as one noted when asked regarding the cause of the disorder: “*I don’t know*. *I didn’t want to treat it because I was scared of those people when they have seizure*” (female, 74 years).

Each additional year of formal education received by healers was associated with lower odds of treating seizure disorders (OR: 0.83 p = 0.017). Each additional patient a healer saw in the previous week was associated with greater odds of treating seizure disorder (OR: 1.10 p = 0.033). Gender, type of healer, and immigration status were not associated with different treatment practices (Table 2).

**Table 2:** Odds of treating seizure disorders (xi-Tsonga name: *Mavabyi ya ku wa*).

	Odds Ratio	Standard Errors	95% CI
Sex (female)	0.47	0.25	0.17–1.31
Age category (>median age)	1.02	0.02	0.99–1.06
Education (per additional year)	0.83	0.07	0.71–0.97*
Moved from Mozambique	1.16	0.53	0.47–2.85
Type of healer (Inyanga)	0.74	0.33	0.31–1.77
Number of patients (per each additional)	1.10	0.05	1.01–1.20*

*statistically significant: p-value <0.05

#### Losing touch with reality (xi-Tsonga *name*: *Nhlanyi)*

Forty-seven percent of healers believed they could successfully treat an adult who had lost touch with reality (xi-Tsonga name: Nhlanyi) There was agreement among healers that this condition could manifest itself differently among patients, but one healer best described the symptoms of *Nhlanyi*: “You will see him/her counting numbers that doesn’t exist, laughing and do[ing] things that are not normal; they differ on their illnesses. There are some those when they are mental ill they will be running around, some will walk naked and some will always be laughing (woman, 45 years). Healers explained the cause of the illness to be a source outside the patient: “*Some you find that they have demons and [they] are the ones that are making her to be [mentally] ill*. *And then if you give her treatment for the calling*, *you find that she will be cured*” (female, 37 years). Others conveyed that while they are able to minimize associated behaviors and psychosis, they may not always be able to cure the disorder: “*When a person comes being mentally ill*, *you find that he is running around … I am able to give him treatment that will help him to have less power and he will stop fighting people*. *Then when I realize that I am failing to treat him*, *I will take him to hospital*” (male, 31 years). Herbal remidies were delivered via drops into the patient ears and eyes to ensure the treatment directly reached the brain. Female healers demonstrated lower odds of treating *Nhlanyi* than their male counterparts (OR: 0.26 p = 0.024), while those who had emigrated from Mozambique had greater odds of treating this illness (OR: 2.86 p = 0.032). Each additional patient a healer cared for was associated with higher odds for treating *Nhlanyi* (OR: 1.17 p = 0.010). Formal education was not associated with willingness to treat *Nhlanyi* (Table 3).

**Table 3:** Odds of treating some who has lost touch with reality (*Nhlanyi)*.

	Odds Ratio	Standard Errors	95% CI
Sex (Female)	0.26	0.16	0.08–0.84*
Age Category (>median age)	1.01	0.02	0.98–1.05
Education (per additional year)	0.88	0.07	0.74–1.03
Moved from Mozambique	2.86	1.40	1.09–7.47*
Type of healer (Inyanga)	1.02	0.47	0.41–2.53
Number of patients (per each additional)	1.17	0.07	1.04–1.31*

*statistically significant: p-value <0.05

#### Paralysis on one side of the body (xi-Tsong name: *Ku Oma Rihlanguti*)

Fifty-nine percent of healers believed they could successfully treat an adult patient who had experienced a paralyses on one side of the body (xi-Tsong: *Ku Oma Rihlanguti; Table 4*). None of the healer characteristics examined were found to have a statistically significant association with difference in odds for treating paralysis symptoms (Table 5). Paralysis on one side of the body was thought to either be caused by “*a nerve that has blocked the movement of blood”* (female, 54 years) or *“by thinking a lot…the nerves on the head get tired and start to ache; you will be affected by stress and it will be followed by Ku Oma Rihlanguti*” (male 59 years). The traditional solution was to “*cut that nerve…*. *Then we are able to trick the ku oma rihlanguti not to continue and attack other nerves in the body and she will be cured on the paralyzed side*.” (female, 54 years). This same healer explained the challenge of working with Ku Oma Rihlanguti patients who go to the health clinic, “*they are doing physiotherapy*, *injecting and give them tablets but she will never be cured*. *She is going to walk because they are training her but it will not be the same as when she is at the traditional healer … It’s about consulting early to us because if you went to the clinic and they inject you for a long time*, *it is hard to treat you and get well*” (female, 54 years).

**Table 4:** Odds of treating paralysis on one side of the body (*Ku Oma Rihlanguti*).

	Odds Ratio	Standard Errors	95% CI
Sex (Female)	0.36	0.21	0.12–1.12
Age Category (>median age)	1.02	0.02	0.99–1.05
Education (per additional year)	0.87	0.07	0.74–1.01
Moved from Mozambique	1.13	0.54	0.44–2.88
Type of healer (Inyanga)	2.26	1.02	0.93–5.48
Number of patients (per each additional)	1.10	0.06	0.99–1.22

**Table 5:** Odds of treating substance use.

	Odds Ratio	Standard Errors	95% CI
Sex (Female)	0.25	0.14	0.08–0.74*
Age Category (>median age)	0.99	0.02	0.96–1.03
Education (per additional year)	0.81	0.08	0.66–0.98*
Moved from Mozambique	0.56	0.32	0.18–1.73
Type of healer (Inyanga)	1.39	0.77	0.47–4.10
Number of patients (per each additional)	1.07	0.04	0.98–1.16

*statistically significant: p-value <0.05

#### Substance use

Twenty-one percent of healers believed they could treat an adult patient with active substance abuse. Healers were hesitant to treat patients who used drugs or alcohol, believing that the patient will continue to use drugs or alcohol regardless of the treatment provided. Female healers were less likely to treat substance abuse disorders (OR: 0.25 p = 0.012), while each additional year of education completed was associated with lower odds of treating substance abuse (OR: 0.81 p = 0.032). No significant association was found between a healer’s age, healer type, immigration status, or the number of patients they attended to in a week and their odds for treating substance abuse disorders (Table 5).

#### Cost for treatment

According to interview responses regarding cost for services provided to adults, median reported fees charged for treating (i) substance abuse was 500 South African Rand (~US$35), (ii) 1000 Rand (~US$70) for treating seizure disorders and paralysis on half of the body, and (iii) 1500 Rand (~US$105) to treat patients who have lost touch with reality. Many healers see their profession as not only important, but lucrative: “*In the past there was no money when you are a healer but nowadays it’s better because there is money … and they are very expensive when they charge*” (female, 75 years). Another healer described an incentive to treat patients while not charging more than necessary, “*If I have treated someone*, *his relative will tell other people that their patient has been cured then I will get more money after that*. *They told us in our organization not to overcharge a person*” (male, 78 years). Healers often recognize the financial constraints of poorer patients, and may choose to lower fees or accept payment(s) in-kind; as one healer noted, “*Maybe you come to the healer with a blanket*, *they will allow it because [our ancestors] were not after money and we are following their footsteps*,” and patients may even “*pay by credit until you finish your installment*” (female, unknown age). Another healer supported this notion: “*This illness is very expensive because not everyone is able to treat and cure them*, *I can charge R1500 but when you don’t have it we don’t force you to pay because we want you to be cured*” (female, 37 years). Many healers acknowledge that treating mental disorders is challenging, and some only charge patients if and when their illness has been cured, as one stated, “*I don’t charge a patient if he is not cured*” (male, 31 years).

#### Referral to allopathic care

Some healers perceive the importance of allopathic care and report initially referring (at least adult) patients to the clinic to be tested for HIV before they treat them. As one explained, “*We don’t give treatment to the patient when she arrives but we have to refer her to the clinic to be tested [for] everything … if they say [she] needs Western treatment then we will not give the patient our treatments*. *Then we allow the hospital to do their job and when they are done then the patient can come to us to be treated [for] illnesses that are related to traditional*” (female, unknown age). Another healer explained the difficulty that, “*According to the law from Kukula association [the local Bushbuckridge traditional healers’ association] says we have to refer the patient to the clinic but I didn’t refer anyone because I didn’t have the forms to refer the patient with*” (male, 78 years). Many other healers suggested that relying on allopathic medicine for mental illness is fruitless, as treatments from clinicians would at best only control symptoms but never cure the disorders, which they believe could only be achieved through traditional care.

## Discussion

About half of the traditional healers in the Bushbuckridge region of South Africa were confident in their ability to treat seizure disorders, patients who had lost touch with reality, and paralysis. Health care providers have reason to be concerned, given the potential negative impact on the prognosis of patients who delay or interrupt allopathic services. Patients with untreated seizure disorders risk severe brain injury [37]depression [38] and increased risk of mortality [39,40], while often being restricted from participating in normal daily activities, including school or employment. Given that capacity to effectively control many seizure disorders exists locally at no cost (specifically treatment for epilepsy), patients seeking traditional treatment for uncontrolled seizure disorders may be delaying effective biomedical treatment.

The provision of traditional treatment of patients who have lost touch with reality is also concerning, as they may be suffering from psychosis, dementia, or bipolar depression. Early identification and treatment could improve the lives of many of these patients [40] as these illnesses are associated with high risk of early mortality[41–43], disability[44,45], and decreased quality of life and depression[46,47] [48]. Finally, the delay in care seeking among people who have experienced paralysis could result in additional risk of sensory disturbance and vertigo/dizziness if the symptoms were caused by a stroke and increase the risk of mortality [49,50] and reduced quality of life among survivors who do not receive effective rehabilitation[51].

Considering the low median income and the accessibility of free primary health care, the cost of healers’ traditional treatment is comparably high [52]. A recent South African study on cost for epilepsy treatment found that compared to allopathic care, the costs for traditional medicine was appreciably higher and varied in terms of cost by patient, with one patient reporting to only have to pay if the epilepsy is cured [53]. Our study and other research conducted in the Bushbuckridge area lead us to presume that many local patients with MNS symptoms do seek out, accept and pay for traditional treatments [17]. While our study did not address the question of healer qualifications or success in reducing or eliminating patient symptoms, a recent systematic review suggests healer treatment may improve acute relapses of major mental illnesses such as schizophrenia and bipolar disorder, but has little impact on the long term progression of the disease [54]. In contrast, common mental disorders, including depression, anxiety, and social difficulties seem more likely to respond to traditional treatments [54].

Approximately 20% of healers reported treating substance abuse (alcohol and drugs). Given the high prevalence of alcohol in the rural South African context [55,56], the hesitancy of healers to provide treatment for these conditions is surprising. Interestingly, there is little allopathic treatment for substance abuse in rural Bushbuckridge, perhaps indicating local perception that this behavior cannot be modified. Healers expressed the belief that patients with substance abuse problems could only be treated if they expressed the desire stop abusing drugs or alcohol; few community members appear to seek traditional support for substance abuse issues.

While our study found Inyanga healers more likely to treat paralysis and substance abuse, they have also been found to be less likely than other healers to refer patients with mental health concerns to allopathic care [57]. A recent study among South African traditional healers found healer attitudes, perceived behavioral control, and past referral behavior to be predictive of healers’ intention to refer mentally ill patients to allopathic care [57]. South African healers are, in fact, already routinely and often successfully partnering with the biomedical health system to care for patients suspected of or known to have HIV and/or TB, especially in providing such patients referral services to clinical centers, which the government requires and healers are extremely cautious of breaking this regulation [58]. This referral process remains informal and unrecorded. Yet competency with and completion of the referral process remain an important skill and behavior, and transferring this to the identification of patients with MNS disorders is both practicable and socially acceptable [58].

While our study allows for potential extrapolation of results to the region’s broader traditional practitioner population, we also recognize potential limitations of our findings. We do not know how healer diagnoses translate to allopathic categories of disease. Healers would often volunteer the corresponding allopathic name for the traditional illness, but there was no way to verify that their perception was accurate. Because of this limitation we have used illnesses descriptions or the xi-Tsonga name for illnesses described by the healers, not the biomedical name. While our sample was a random sample of traditional healers in the Kukula organization, we recognize that our sample may not reflect the behavior of healers outside of Bushbuckridge. Healers’ memory from the previous month of the number of patients treated, diagnoses and treatments given, payments received and treatment outcomes may all have been affected by recall bias. Furthermore, it possible is that social desirability bias influenced some healers to under report their treatment of MNS-related disorders, if concerned about criticism from the biomedical system. Attempts were made to reduce the potential for this bias by having the interviewer inform participating healers that responses would not be reported to local or other government or health authorities. Finally, this study recruited participants who were members of the Kukula association. There are likely some traditional healers in the area who are not members of this association but we have no data about these individuals.

## Conclusion

In rural northeastern South Africa many practicing traditional healers are continuing to see patients and offer treatments for MNS disorders and related symptoms, and often charge high fees compared to the otherwise free or reduced costs of care at public health facilities. While healers were able to link traditionally defined mental illness to allopathic diagnoses, about half were confident in their ability to cure seizure disorders, patients who have lost touch with reality, and paralysis on one side of the body. Given the negative long-term health implications of delayed health seeking among people suffering with any of these conditions, there is a significant need to better understand areas for possible collaboration in the care of patients with MNS illnesses. Through identifying effective and affordable MNS treatments, promoting applicable communication strategies between the systems, and supporting practitioners within both systems to offer patients the best-suited therapies, treatment and management of MNS illnesses can be vastly improved in this region, the burden of disease reduced and patients’ quality of life improved.
